# The problem of choice

**DOI:** 10.1186/1476-4598-7-86

**Published:** 2008-11-23

**Authors:** Hassan R Naqvi, Shawn Mathur, David Covarrubias, Josephine A Curcio, Christian Schmidt

**Affiliations:** 1 The Cleveland Clinic Foundation, 9500 Euclid Avenue, Cleveland, Ohio, 44195, USA; 2 Section of Molecular Genetics and Microbiology, University of Texas, 1 University Station, A 5000, Austin, TX 78712, USA; 3 Institute for Cellular and Molecular Biology, University of Texas, 1 University Station, A 5000, Austin, TX 78712, USA; 4 Molecular Cancer, Biomed Central Ltd., Middlesex House, 34–42 Cleveland Street, London W1T 4LB, UK

## Abstract

Convictions are a driving force for actions. Considering that every individual has a different set of convictions and larger groups act once a consensus decision is reached, one can see that debate is an inherent exercise in decision-making. This requires a sustainably generated surplus to allow time for intellectual exchange, gathering of information and dissemination of findings. It is essential that the full spectrum of options remain treated equally. At the end of this process, a choice has to be made. Looking back at a later time point, a retrospective analysis sometimes reveals that the choice was neither completely free nor a truly conscious one. Leaving the issue of consequences of a once made decision aside, we wish to contribute to the debate of the problem of choice.

## Discussion

Scientific publications are well known for using hedging [1], a writing resource whereby conclusions are written with precision, caution and due deference to the prevailing opinion in anticipation of possible opposition to claims made. As it is commonly accepted practice in science, every claim must be based on experimental evidence, shared via peer-reviewed articles, thus, enabling readers to 'trace' arguments/theses to an experimental source. One can see that emotional outbreaks are not helpful in advancing science and refining our understanding of a given problem. However, research priorities, their presentation and even their criticism is informed by convictions which melds the conclusions from data sets in a particular manner and also directs the criticism generated against these conclusions. They provide a framework, a set of principles in the absence of absolutes in scientific research. These convictions when based on years of experience, survey of literature and counseling of peers with in the field are useful in furthering research. Even in the face of contrary, and seemingly divergent data, convictions informed by knowledge and wisdom enables the investigator to sense and grab on to that fine thread of logic that affirms seemingly opposing pieces of evidence and leads the way to the discovery of new and wondrous phenomena.

At the same time, convictions also drive a sense of productive doubt with results or claims that seem far removed from expectation. While questioning the results and the methodology is an effective way to think about alternative ways to deal with problems, they however do not constitute a valid basis to lend credibility to alternate convictions. This is especially true in cases where the skeptic lacks a record of demonstrable scientific data that would support their point of view. Exceptions to principles and/or logical gaps in a particular model do not form the basis for absolute rejection and scrapping of a theory or the confirmation of an alternate point of view. The current debates dealing with global warming and evolution may serve as cases in point. While there is sound evidence for both global warming and evolution, gaps in the body of evidence neither constitute grounds for disavowing the entire body of evidence nor giving credence to the opposing point of view in the absence of scholarly peer-reviewed data.

Scholarly communication should embrace every viewpoint of a given data set, transformed as testable thesis, ideally as a peer-reviewed and published article, freely available and stored appropriately. Other aspects of this process are introduced, extended and discussed elsewhere [2-8], resulting in a concerted effort to advance knowledge, independent of the location of a given scholar. Contrary to the harmony in science, the modus operandi, often seen in the process of decision making in larger groups, simplistically defined as politics [9], can be described in its essence as 'divide and rule' [10] along with varying degrees of populism [11]. Given the challenges in addressing issues such as global warming, world hunger and poverty, inductive reasoning forbids the division of talents and resources by application of the 'divide and rule' doctrine. Concordantly, citizens are encouraged to strive for a harmony in government, allowing the community to grow, bloom and withstand significant challenges and changes. In the opinion of the authors, no other profession is subjected to profound challenges and changes as science; harmony increases productivity and frees talent, all for the good of the greater community. We, therefore wish to initiate a debate about the problem of choice.

## Conclusion

Conviction by itself without any basis reeks of autocracy, and harmony for the mere desire of harmony might be taken as weakness, and debate without actual data can be seen as argumentative. Harmony, driven by conviction, which itself is based on vetted and debated data, is the way forward.

## Competing interests

HRN, DC, SM and JAC declare that there are no competing interests. CS is deputy editor of Molecular Cancer and receives no remuneration for his efforts.

## Authors' contributions

CS drafted and finalized this paper. HRN discussed ideas with CS that ultimately resulted in this paper and provided insightful critique. DC, SM and JAC assisted in gathering background information.
